# Decreased intersubject synchrony in dynamic valence ratings of sad movie contents in dysphoric individuals

**DOI:** 10.1038/s41598-021-93825-1

**Published:** 2021-07-13

**Authors:** Xueqiao Li, Yongjie Zhu, Elisa Vuoriainen, Chaoxiong Ye, Piia Astikainen

**Affiliations:** 1 Department of Psychology, University of Jyvaskyla, P.O. Box 35, 40014 Jyväskylä, Finland; 2 Department of Computer Science, University of Helsinki, 00014 Helsinki, Finland; 3 Human Information Processing Laboratory, Faculty of Social Sciences/Psychology, Tampere University, 33014 Tampere, Finland; 4 Institute of Brain and Psychological Sciences, Sichuan Normal University, Chengdu, 610000 China

**Keywords:** Human behaviour, Depression

## Abstract

Emotional reactions to movies are typically similar between people. However, depressive symptoms decrease synchrony in brain responses. Less is known about the effect of depressive symptoms on intersubject synchrony in conscious stimulus-related processing. In this study, we presented amusing, sad and fearful movie clips to dysphoric individuals (those with elevated depressive symptoms) and control participants to dynamically rate the clips’ valences (positive vs. negative). We analysed both the valence ratings’ mean values and intersubject correlation (ISC). We used electrodermal activity (EDA) to complement the measurement in a separate session. There were no group differences in either the EDA or mean valence rating values for each movie type. As expected, the valence ratings’ ISC was lower in the dysphoric than the control group, specifically for the sad movie clips. In addition, there was a negative relationship between the valence ratings’ ISC and depressive symptoms for sad movie clips in the full sample. The results are discussed in the context of the negative attentional bias in depression. The findings extend previous brain activity results of ISC by showing that depressive symptoms also increase variance in conscious ratings of valence of stimuli in a mood-congruent manner.

## Introduction

Depressive disorder is a mental health disorder that causes patients to exhibit a unique combination of affective, cognitive and somatic symptoms^1^. According to cognitive theories of depression^2,3^, depressive disorder is associated with an attentive negative bias in information processing that causes individuals to exhibit selective attention to dysphoric stimuli, ruminate on self-referential depressive thoughts and recall sad memories.

Several empirical studies support the presence of attentive negative processing bias in individuals with pre-clinical and clinical depression. Studies using reaction time paradigms, such as the dot-probe task and emotional Stroop task, have consistently reported a negative bias towards sad faces and other dysphoric contents in these individuals^4,5^. Eye tracking studies have similarly demonstrated increases in gaze maintenance on dysphoric stimuli in pre-clinical and clinical depression cases^6,7^. Studies using event-related potentials have revealed a depression-related negative bias in emotional face processing^8–11^. Depressed participants also rate neutral faces as sad more often than controls^12,13^. However, naturalistic stimuli rarely appear in depression studies on negative bias, although they would allow a more ecologically valid approach than, for example, the presentation of static pictures.

In response, in this study we investigate whether depressive symptoms affect the dynamic valence ratings of emotional movie clips. We are particularly interested in the ratings’ intersubject correlation^14,15^ (ISC), which reflects how similarly people evaluate stimulus content. Group differences in ISC, specifically when viewing sad movies, can be expected due to depression-related attentive bias^2,3^. Previous studies have not investigated the ISC of emotional movie contents’ dynamic valence ratings neither in healthy nor depressive participants. However, for healthy participants, some studies have investigated valence rating ISC for the subjective emotions that naturalistic stimuli elicited^16^, and for eye movements during movie watching^17^.

Findings from brain activity measurements indicate that, in healthy individuals, brain activity patterns during movie watching are surprisingly similar across participants^18–27^. ISC in neural responses has been found to be affected by, for instance, valence and arousal from emotional events^20^, attentional engagement with stimuli^23–25^ and emotional involvement^23^ regarding the stimuli. In individuals with neuropsychiatric disorders, meanwhile, within-group neural synchrony during natural stimulation seems to be lower^28–34^. Studies have reported weaker temporal synchronization compared to neurotypical controls in participants with autism^28,29^, schizophrenia^31,32^ and major depressive disorder (MDD)^33,34^. When depressed participants viewed films with negative emotional valence, they exhibited weaker fMRI response synchronization as well in several neural networks associated with sensory and emotional functions and attention, as compared to controls^33^. Furthermore, in adolescents, those who had more severe depressive symptoms exhibited less similar fMRI responses to an emotional movie clip^34^.

With this study, we complement previous brain activity findings^33,34^ by investigating dysphoric participants’ (individuals with elevated depressive symptoms) dynamic valence rating ISC for emotional movie clips in comparison to controls. We anticipated lower valence rating ISC in the dysphoric rather than the control group based on previous findings demonstrating decreased ISC for neural activity in non-neurotypical samples^33,34^.

We utilized amusing, fearful and sad movie clips as stimuli. We expected lower ISC in the dysphoric group particularly for sad movie clips because of the mood-congruent attentive bias associated with depression^8–11^. Amusing movie clips were selected to study whether ISC decreased for positive valence stimuli as well, while fearful movie clips were used to study whether ISC decreased for negative stimuli in general or specifically for mood-congruent (sad) stimuli. Cognitive theories of depression suggest that depression-related negative schemata activate negative self-referential information, automatic thoughts and memories, which produce unique and subjective emotional experiences^35^. These can affect how movie content valence is evaluated. Since the activation of depression-related negative schemata is likely to cause individual variability in reactions, sad movie clips could evoke more heterogenous activity (i.e. lower ISC) in the dysphoric group than in the control group.

Previous studies have shown that stimuli with high emotional arousal attract participants’ attention^36^. Therefore, to examine emotional arousal to movie clips, we applied electrodermal activity (EDA) to measure the participants’ phasic responses during the viewing as well^37^. These data can inform us in our interpretation of the valence rating ISC results.

## Results

First, we report the mean values of the EDA responses and dynamic behavioural ratings to amusing, fearful and sad movie clips, and compare the responses between the dysphoric and control participants. Then, we outline the comparison of the dynamic behavioural ratings’ ISC for emotional movie clips in the dysphoric and control groups.

### Electrodermal activity (EDA)

#### Mean phasic EDA values

**Figure 1:**
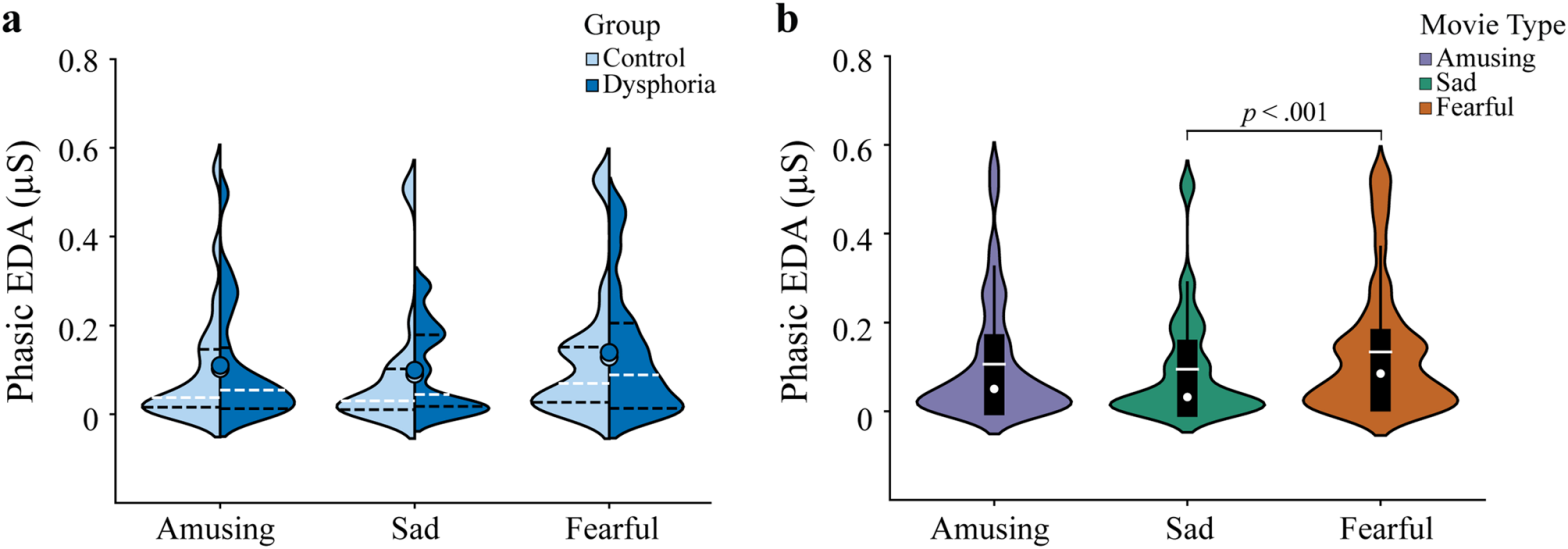
Violin plots for the mean phasic EDA values averaged over the timepoints separately for each movie type. For both (**a,b)**, violin plot outlines illustrate the distribution of EDA estimates using kernel probability density with the bandwidth of 0.2. Wider area of the violin plot represents a higher probability of the values that the EDA take on, and the thinner area corresponds to a lower probability. Please note that the negative values in the violin plot are the estimations of values of the EDA data, which is caused by the use of kernel density estimation. (**a**) The mean phasic EDA values to each movie type in each group (no significant group differences). Each side of the violin corresponds to a different group. Dots denote mean of the EDA. Horizontal white dotted lines show median of the EDA and horizontal black dot lines represent the interquartile range (IQR) of the EDA. (**b**) The mean phasic EDA values to each movie type averaged over the control and dysphoric groups. White dots on the violin plots show the median of the EDA and horizontal white lines represent the mean of the EDA. The black bars in the centre of violins denote the IQR of the EDA. The black lines stretched from the bars show the lower and upper adjacent values (1.5 times the IQR) of the EDA, respectively.

For the mean phasic EDA values in response to emotional movie clips, the analysis of variance (ANOVA; Movie type × Group) showed a main effect for Movie type (*F*(2,40) = 8.26, *p* = 0.001, ${\mathrm{\eta }}_{\mathrm{p}}^{2}$ = 0.292). Paired samples t-tests showed a larger amplitude in phasic EDA to fearful (*M* = 0.134 µS, *SD* = 0.145) than sad (*M* = 0.095 µS, *SD* = 0.124) movie clips (*t*(42) = 3.78, *p* < 0.001, 95% confidence interval (CI) [− 0.060, − 0.018], d = 0.29). When comparing amusing (*M* = 0.106 µS, *SD* = 0.135) and fearful or amusing and sad movie clips, there were no differences between clips (*p* > 0.095, 95% CI [− 0.056, 0.004] and *p* > 0.156, 95% CI [− 0.005, 0.033], respectively). There were no main effects for Group or interaction effects for Group × Movie type, with all *p*-values > 0.818. Figure 1 illustrates the mean phasic EDA values during emotional movie watching.

Because a few participants also had anxiety symptoms, we investigated if the main effect of Movie type reflecting the largest EDA amplitudes to fearful movie clips was explained by anxiety symptoms. We thus conducted an analysis of covariance (ANCOVA; Movie type × Group) controlling for anxiety (Anxiety in Depression, Anxiety, Stress Scales, or DASS-A, scores) symptoms. The main effect of Movie type remained significant (*F*(2,39) = 10.36, *p* < 0.001, ${\mathrm{\eta }}_{\mathrm{p}}^{2}$ = 0.347), suggesting that anxiety symptoms did not explain the EDA to fearful movie clips. There was also no main effect for Group or an interaction effect for Group × Movie type (*p* > 0.117).

#### Correlations

There were no correlations between phasic EDA and Beck’s Depression Inventory-II (BDI-II) scores for any of the movie types in the control group (all *p*-values > 0.736, False discovery rate (FDR) corrected), the dysphoric group (all *p*-values > 0.999, FDR corrected) or the whole sample (all *p*-values > 0.999, FDR corrected).

### Behavioural valence rating

#### Mean valence rating values

**Figure 2:**
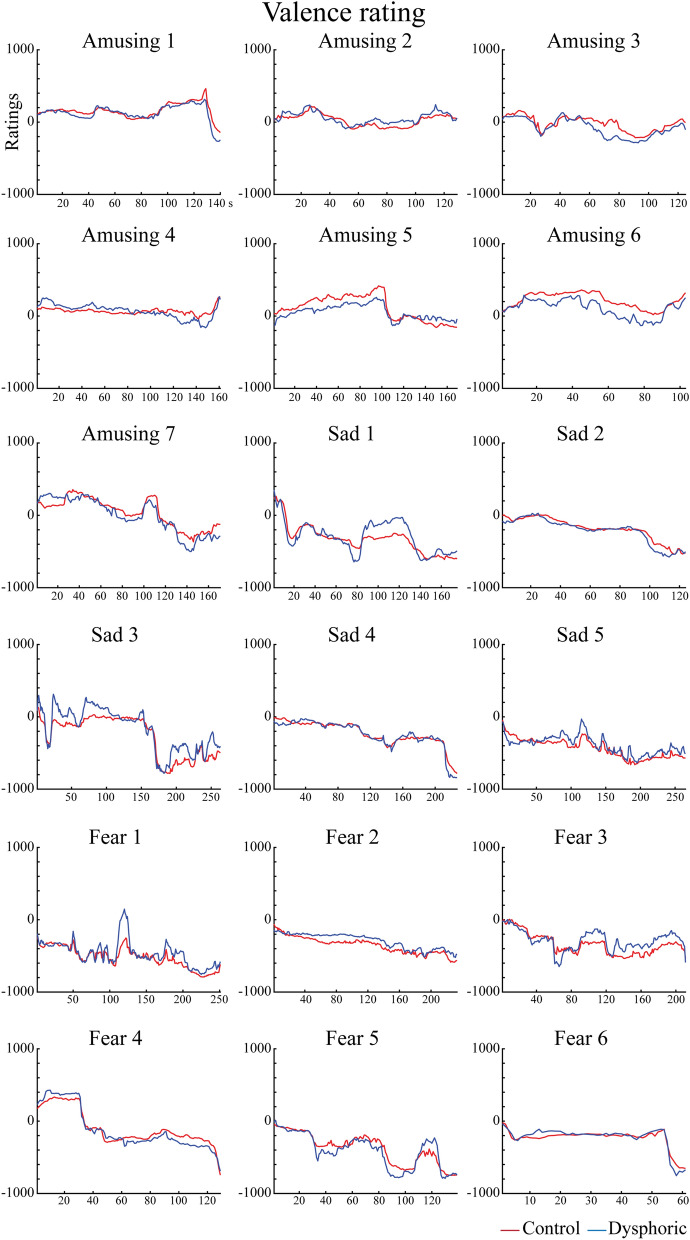
Dynamic valence ratings separately for each movie and research group. Here the ratings range from −1000 to 1000 on an artificial scale. Data of movie Amusing 3 were excluded for the statistical analysis of the EDA responses and valence ratings, because the average value over the timepoints for valence for this amusing movie clip was negative.

**Figure 3:**
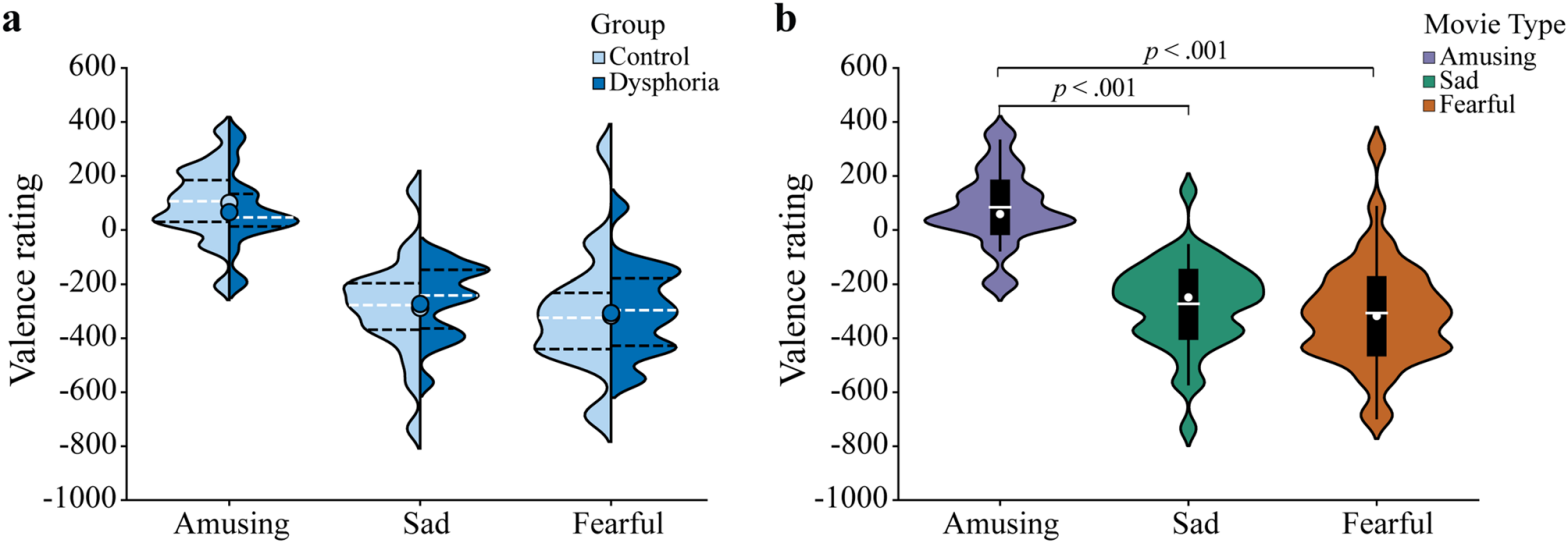
Violin plots for the valence ratings averaged over the timepoints separately for each movie type. For both (**a,b)**, violin plot outlines illustrate the distribution of valence rating estimates using kernel probability density with the bandwidth of 0.2. Wider area of the violin plot represents a higher probability of the values that the valence ratings take on, and the thinner area corresponds to a lower probability. (**a**) The mean valence rating values to each movie type in each group (no significant group differences). Each side of the violin corresponds to a different group. Dots denote means of the valence rating. Horizontal white dotted lines show median of the valence rating and horizontal black dot lines represent the interquartile range (IQR) of the valence rating. (**b**) The mean valence rating values to each movie type averaged over the control and dysphoric groups. White dots on the violin plots show the median of the valence rating and horizontal white lines represent the means of the valence rating. The black bars in the centre of violins denote the IQR of the valence rating. The black lines stretched from the bars show the lower and upper adjacent values (1.5 times the IQR) of the valence rating, respectively.

Figure 2 shows the dynamic behavioural evaluations of movie valence separately for each movie and research group. Figure 2 also provides descriptive information on the dynamic changes at the grand-average level. The results of the mean valence rating values are presented in Fig. 3.

For the mean behavioural valence rating values, the ANOVA (Movie type × Group) revealed a main effect for Movie type (*F*(2,37) = 58.36, *p* < 0.001, ${\mathrm{\eta }}_{\mathrm{p}}^{2}$ = 0.759). We conducted pairwise comparisons with Bonferroni correction separately between amusing and sad, amusing and fearful, and sad and fearful movie clips. Amusing (*M* = 84.1, *SD* = 136.2) movie clips were evaluated as more positive than sad (*M* = − 272.8, *SD* = 160.1) movie clips (*t*(39) = 10.90, *p* < 0.001, 95% CI [290.6, 423.0], d = 2.40). Amusing movie clips were also rated more positively than fearful (*M* = − 306.6, *SD* = 191.9) movie clips (*t*(39) = 10.23, *p* < 0.001, 95% CI [313.4, 467.9], d = 2.35). There was no difference between sad and fearful movie clips (*p* > 0.087, 95% CI [– 5.27, 72.89]). The main and interaction effects for Group were not significant (all *p*-values > 0.599).

Next, the ANCOVA (Movie type × Group) controlling for anxiety symptoms (DASS-A) revealed that the main effect of Movie type remained significant (*F*(2,74) = 55.90, *p* < 0.001, ${\mathrm{\eta }}_{\mathrm{p}}^{2}$ = 0.602). There was also no main effect for Group or an interaction effect for Group × Movie type (*p* > 0.757).

#### Behavioural valence rating ISC

**Figure 4:**
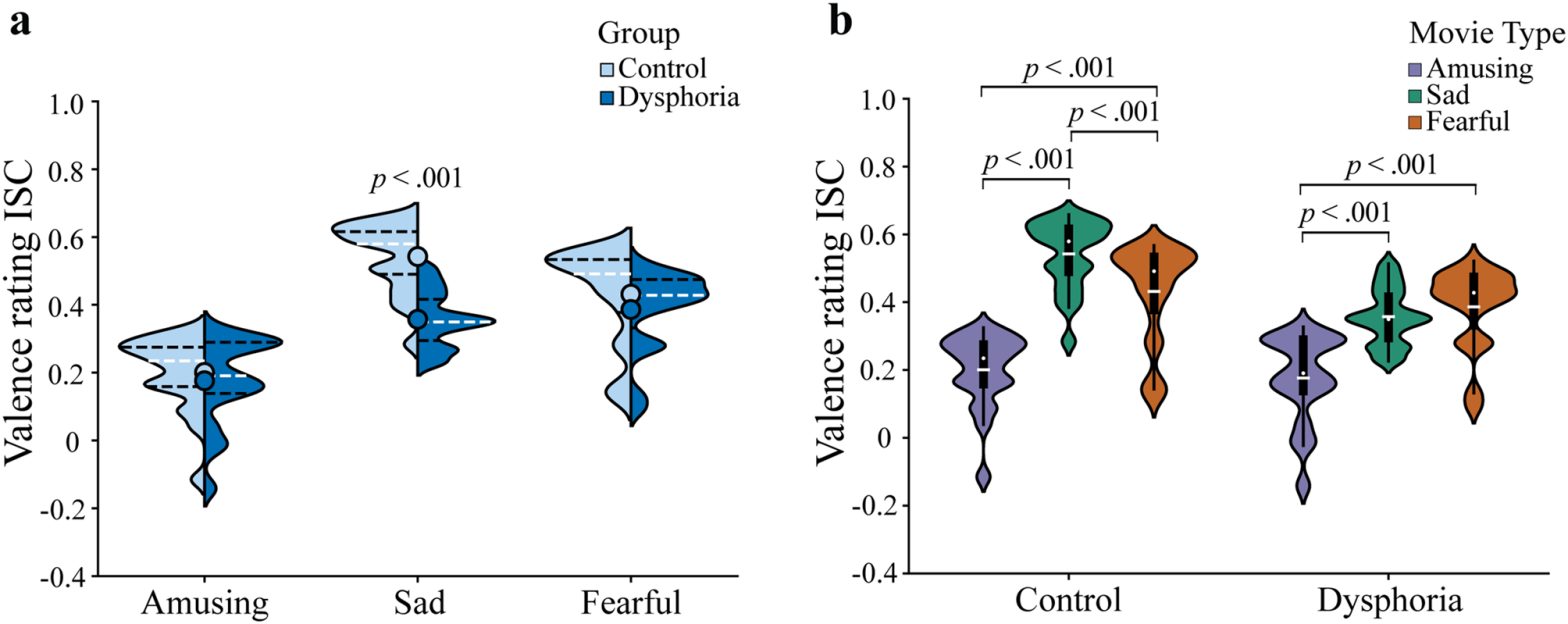
Violin plots for the valence rating ISC for each movie type and research group. For both (**a**,**b)**, violin plot outlines illustrate the distribution of ISC estimates using kernel probability density with the bandwidth of 0.2. Wider area of the violin plot represents a higher probability of the ISC, and the thinner area corresponds to a lower probability. (**a**) Group differences in each movie type are illustrated. Each side of the violin corresponds to a different group. Dots denote means of the ISC. Horizontal white dotted lines show median of the ISC and horizontal black dot lines represent the interquartile range of the ISC. (**b**) Differences of movie type within control and dysphoric group are shown. White dots on the violin plots show the median of the ISC and horizontal white lines represent the means of the ISC. The black bars in the centre of violins denote the interquartile range (IQR) of the ISC. The black lines stretched from the bars show the lower and upper adjacent values (1.5 times the IQR) of the ISC, respectively.

The results of the valence rating ISC are illustrated in Fig. 4.

For valence rating ISC, a repeated-measures ANOVA (Movie type × Group) showed a main effect for Movie type (*F*(2,76) = 97.20, *p* < 0.001, ${\eta }_{p}^{2}$ = 0.719) and Group (*F*(1,38) = 8.70, *p* = 0.005, ${\eta }_{p}^{2}$ = 0.186). The main effects were modulated by an interaction effect between Movie type and Group (*F*(2,76) = 9.38, *p* < 0.001, ${\eta }_{p}^{2}$ = 0.198). Independent *t*-tests with Bonferroni correction investigated this interaction effect by comparing the dysphoric and control groups’ responses to the amusing, sad and fearful movie clips. This demonstrated a significant group difference in ISC for sad movie clips (*t*(38) = 6.23, *p* < 0.001, 95% CI [0.125, 0.245], d = 2.02). The ISC was also larger in the control group (*M* = 0.542, *SD* = 0.102) than in the dysphoric group (*M* = 0.357, *SD* = 0.084). There were no differences in ISC for amusing and fearful movie clips between the dysphoric and control groups (all *p*-values > 0.295).

Next, we compared valence rating ISC for the amusing, sad and fearful movie clips in separate repeated ANOVA within the dysphoric and control groups. For the dysphoric group, the main effect of Movie type was significant (*F*(2,36) = 25.83, *p* < 0.001, ${\eta }_{p}^{2}$ = 0.589). The effect of anxiety symptoms was also investigated in the dysphoric group, though the ANCOVA with DASS-A as a covariate showed no significant Movie type × DASS-A interaction in the dysphoric group (*p* = 0.348). An ANCOVA with Medication as a covariate also showed no significant interaction between Movie type and Medication (*p* = 0.058) in the dysphoric group. Follow-up paired-samples *t*-tests with Bonferroni correction showed that the dysphoric group had larger ISC in the behavioural ratings of sad movie clips (*M* = 0.357, *SD* = 0.084) than amusing clips (*M* = 0.176, *SD* = 0.130; *t*(18) = 5.35, *p* < 0.001, 95% CI [− 0.252, − 0.110], d = 1.65). The behavioural rating ISC for fearful movie clips (*M* = 0.386, *SD* = 0.120) was also larger than for amusing clips in the dysphoric group (*t*(18) = 6.47, *p* < 0.001, 95% CI [− 0.278, − 0.142], d = 1.68). No difference in ISC emerged between sad and fearful movie clips (*p* = 0.323).

For the control group, a repeated-measures ANOVA showed a main effect for Movie type (*F*(2,40) = 93.32, *p* < 0.001, ${\eta }_{p}^{2}$ = 0.824). Subsequent t-tests with Bonferroni correction indicated that the control group had a larger valence rating ISC for sad move clips (*M* = 0.542, *SD* = 0.102) than for amusing ones (*M* = 0.201, *SD* = 0.108; *t*(20) = 15.56, *p* < 0.001, 95% CI [− 0.388, − 0.296], d = 3.25). The controls also had a larger valence rating ISC for fearful movie clips (*M* = 0.432, *SD* = 0.146) than for amusing clips (*t*(20) = 7.10, *p* < 0.001, 95% CI [− 0.300, − 0.163], d = 1.80). The valence rating ISC was thus larger for sad movie clips than fearful ones (*t*(20) = 5.46, *p* < 0.001, 95% CI [0.068, 0.153], d = 0.87).

#### Correlations

For the control group, no significant correlations emerged between the mean valence rating values and number of depressive symptoms for any movie type (all *p*-values > 0.629, FDR corrected). For the dysphoric group, there was a negative relationship between the mean valence rating values for amusing movie clips and number of depressive symptoms (r = − 0.639, *p* = 0.003 (FDR corrected *p* = 0.017), 95% CI [− 0.834, − 0.206]): the more depressive symptoms the participants had, the less positive they evaluated the amusing movie clips. There were no correlations between BDI-II scores and mean valence rating values for sad and fearful movie clips (all *p*-values > 0.999, FDR corrected). For the whole sample, Spearman’s rank correlation test showed no correlations between the valence ratings and number of depressive symptoms for any movie type (all *p*-values > 0.505, FDR corrected).

The correlations between valence rating ISC and BDI-II scores were non-significant for each movie type in the control group (all *p*-values > 0.955, FDR corrected) and the dysphoric group (all *p*-values > 0.999, FDR corrected). For the whole sample, Spearman’s rank correlation test revealed a negative relationship between valence rating ISC and number of depressive symptoms during the viewing of sad movie clips (r = − 0.552, *p* < 0.001 (FDR corrected *p* < 0.005), 95% CI [− 0.754, − 0.281]): the more depressive symptoms the participants had, the less synchronized they were in their valence ratings of sad movie clips. There were no relationships between valence rating ISC and BDI-II scores for amusing and fearful movie clips (all *p*-values > 0.208, FDR corrected).

## Discussion

In the present study, by applying ISC analysis, we compared dysphoric and non-dysphoric participants’ ISC in their behavioural valence ratings of emotional movie clips. We found that dysphoric participants were less synchronized than controls in their dynamic valence ratings, specifically for sad movie clips.

A previous study showed increased heterogeneity in the synchrony of depressed participants’ fMRI responses to movies in several neural networks associated with sensory and emotional functions, as well as attention^33^. These authors suggested that decreased ISC in the depressed group could be related to their unique and distinct internal processes evoked by the movie clips. This is a plausible explanation in this study as well for the decreased synchrony in the behavioural valence ratings of movies in participants with depressive symptoms. It is also possible that in the control group, the valence ratings were more consistent since they reflected more stimulus-dependent reactions evoked by the movie contents, while the dysphoric group’s ratings could reflect more participant inner processes, such as evoked memories and emotions. The exact mechanism underlying the dysphoric group’s rating desynchronization is unclear, however. It is possible that the memories and emotions evoked by the sad movie clips interrupted their rating behaviour, causing more mind wondering than in the control participants. There could also have been more internal elaborative processing in the dysphoric group, such as intense emotion regulation or rumination^38,39^. In our previous eye tracking study^40^, where participants freely viewed video-recorded emotional and neutral dyadic conversations, the more depressive symptoms the participants had, the less viewing patterns aligned with the conversation flow the correlative analysis showed. This finding reflects depressed participants’ difficulties with attentively following social interactions. Notably, social interactions were also depicted in many of the movie clips in this present study. However, it is unclear whether the results of this study reflect increased synchrony specifically in contents related to social interactions.

Instead, our data demonstrated a depression-related effect specifically in the ISC for sad movie ratings: the ISC was lower in the dysphoric group for sad clips, but no such effect emerged for fearful or amusing clips. In addition, our data showed that for the whole sample, the more depressive symptoms the participants had, the less synchronized they were in their valence ratings of sad movie clips. We expected this based on the mood-congruent attentive bias in individuals with depression^2,3^. Our finding on behavioural movie valence rating ISC is in line with a previous fMRI study showing that participants with MDD had less synchronized fMRI responses compared to control participants when viewing movies with negative emotional valence^33^. However, in that study^33^, negative movie contents were not divided into separate categories (e.g. sad and fearful), but mixed. Concordant with our expectations for sad attentive bias in depression, we did not find any group differences in ISC for fearful movie clips. Indeed, bias towards threatening content is not a common finding in depressive populations, but more associated with anxiety^4,41^ (see, however, Rantanen et al.^42^ for depression-related attentive bias to interpersonally aggressive pictures). For this reason, we did not expect group ISC differences for fearful movie clips.

ISC was also similar in the groups’ ratings of amusing movie clips. Some previous studies have shown less positive expressive behaviour and subjective reports in response to pleasant films and scenes in depressed individuals compared to controls^43,44^; blunted positive emotional reactions in general have also been reported in depression cases^45^. In our study, the participants rated the movie clips’ valence rather than their own subjective emotions. Our results suggest that the ISC of these stimulus-related valence ratings are observable in sad, but not for fearful or amusing, movie clips. It is also notable that the mean dynamic valence rating values did not differ for any of the movie types between the groups, only in ISC.

Previous studies have stated that high emotional stimuli arousal cause attentional capture^36,46^, which increases intersubject synchrony in brain activity^20^. It is thus possible that the present study’s group difference in rating ISC for sad movie clips is explained by different levels of arousal from the different movie types. However, this seems not to be the case in light of the EDA recordings, reflecting emotional arousal^37^, in which no group differences emerged. Additionally, at the whole sample level, a smaller amplitude in phasic EDA for sad movie clips than for fearful ones indicated the participants’ lower arousal from the former.

In both groups, the dynamic valence rating ISC was lowest for the amusing movie clips. A previous study found that a negative stimulus valence is associated with increased ISC in brain activity in the default-mode network and limbic system^20^. In addition, research has suggested that negative emotions urge people to narrow their mental focus for immediate survival benefits, while positive emotions broaden people’s attention and promote play and exploration^47,48^. Therefore, broader attention to amusing movie scenes than to negative-valence scenes, and thus more variable viewing pathways, could explain the lower valence rating ISC for amusing clips.

In general, our results are consistent with previous neuroimaging findings in autism, schizophrenia and melancholic MDD, which have shown less fMRI response synchronization in multiple cortical areas during naturalistic stimulation in these groups compared to neurotypical samples^28–30,32,33^. It is understandable that neurodevelopmental and psychiatric disorders can increase variability between individuals in brain function due to “noisier” neural processes. Here, we demonstrate a similar increase in variability at the behavioural level in conscious ratings of valence of dynamic visual stimuli.

The present study has a few limitations. First, we used silent movie clips without captions. Therefore, the emotions evoked by the clips may not have been as strong as those evoked by clips with captions or background music, speech and other sounds. The missing audio might also have caused difficulties in interpreting the plot, because some of the scenes contained conversations. We did not want to use sounds because spoken non-native language could have confused the participants, and differences in their linguistic abilities could have caused some group differences. Further, captions could have guided spatial attention away from the natural targets of attention in the visual scene. We aimed to counteract the effects of the missing audio by providing the participants a short explanation of the current situation in the movie before showing each clip. Another limitation is that 9 of the 21 dysphoric participants were taking anti-depressant medication during the study, though the covariate analysis did not reveal an effect for medication. Several dysphoric participants also had some anxiety symptoms during the study. The analysis with anxiety symptoms (DASS-A) as a covariate, however, suggested that anxiety did not affect the results. Lastly, although all the selected movie clips were rated for discrete emotional feelings and categorized into amusing, sad and fearful movie types in a previous study^49^, our participants rated them only on a negative–positive valence scale, and categorization was not requested. It is also worth noting that compared to the previous study^49^, we played the movie clips without sound, and some parts of the scenes were cut. Therefore, the original categorizing into emotional categories may not be preserved exactly as in Schaefer et al.^49^.

In conclusion, the present study investigated intersubject synchronization across dysphoric and control participants as they rated the valence of amusing, sad and fearful movie clips. The dynamic valence rating ISC of sad clips was lower in the dysphoric group compared to the control group, most probably reflecting negative attentive bias related to depression symptoms. The findings of the present study provide the first evidence of a reduction in within-group synchrony in behavioural valence ratings due to depressive symptoms.

## Methods

### Participants

The required sample size for the present study was computed with an a priori power analysis using G*Power^50^ (version 3.1.9.2). We selected a repeated-measures ANOVA to test the interaction between a within-subject variable (Movie type) and a between-subject variable (Group). As no previous study has investigated valence rating ISC in depression (or valence rating ISC of dynamic stimuli in general), the sample size in the present study was estimated based on a conventional large effect size^51^ (${\eta }_{p}^{2}$ = 0.14) using the specifications in SPSS (IBM SPSS Statistics; IBM Corporation, NY, USA). Based on this calculation, 19 participants were required for each group (healthy and dysphoric) to achieve a statistical power of (1 − β) = 0.85, a significance level of α = 0.05 and a non-sphericity correction of ε = 1. This sample size is larger than in some previous fMRI studies with clinical populations^28,29^, but similar to the sample sizes in a depression-related study^33^.

In total, 24 healthy (4 males and 20 females) and 23 dysphoric (8 males and 15 females) Finnish-speaking participants were recruited for the study. All participants were recruited through flyers distributed around the Jyväskylä area and via the student email lists of the University of Jyväskylä. The study protocol occurred in accordance with the Declaration of Helsinki and was approved by the Ethics Committee of the University of Jyväskylä. Written informed consent was obtained from all participants before measurement began.

The inclusion criteria were right-handedness, normal vision and hearing ability, and age between 18 and 40 years. Participants who had brain damage, neurological disorders or a history of drug or alcohol abuse were excluded. Another exclusion criterion was current or previous psychiatric disorders and symptoms, except for depression and anxiety symptoms in the dysphoric group. Depressive symptoms were measured with a self-report inventory of depressive symptoms, the BDI-II^52^. Participants with BDI-II scores of 14 or higher were included in the dysphoric group, and participants with BDI-II scores less than 10 were included in the control group.

Some, but not all, of the dysphoric participants had current depression diagnoses. Seven participants were interviewed by a medical doctor independent of the study, and their depression diagnoses were confirmed at the beginning of the study. Eight participants self-reported having a current diagnosis of depression. Six dysphoric participants did not have a depression diagnosis. For all dysphoric participants, the BDI-II score range was 16–45.

One dysphoric participant was excluded due to failure to meet the inclusion criteria, and 2 controls and 1 dysphoric participant could not be included in the final sample due to artefacts in their psychophysiological recordings. Therefore, 22 control (4 males and 18 females) and 21 dysphoric (7 males and 14 females) participants were included in the EDA data analysis. For the behavioural ratings, the data of 3 more participants were lost due to a technical problem. Thus, in the final sample for the analysis of behavioural ratings, there were 21 controls (4 males and 17 females, age range 19–40 years, *M* = 26.86 years, *SD* = 6.64, BDI-II scores range 0–5) and 19 dysphoric participants (7 males and 12 females, age range 18–40 years, *M* = 30.00 years, *SD* = 7.65, BDI-II scores range 16–45). The participants’ demographic and clinical information for the EDA analysis is presented in Table 1.

**Table 1:** Demographic and clinical information for each group. Statistics present independent t-test or *x*^2^ test values investigating the group differences.

Characteristics		DYS (n = 21)	CTRL (n = 22)	Statistics
Age (year)	Mean	30.29	26.68	*t*(41) = 1.692, *p* = .098
	SD (range)	7.42 (18–40)	6.54 (19–40)	
Level of Education^a^	Low	1	0	*x*^2^(2) = 4.410*, p* = .110
	Medium	11	6	
	High	9	16	
Gender	Male	7	4	*x*^2^(1) = 1.296*, p* = .255
	Female	14	18	
DASS-A	Mean	9.76	1.50	*t*(41) = 4.829, *p* < .001
	SD (range)	7.58 (1–38)	2.58 (0–10)	
BDI-II	Mean	29.43	2.00	*t*(41) = 15.315, *p* < .001
	SD (range)	8.02 (16–45)	1.77 (0–5)	
Severity of Depression^b^	No diagnoses	6	Na	Na
	Mild (F32.0)	0	Na	Na
	Moderate (F32.1)	6	Na	Na
	Recurrent moderate (F33.1)	1	Na	Na
	Severe (F32.2)	1	Na	Na
	Recurrent severe (F33.2)	4	Na	Na
	Severity unknown	3	Na	Na
Depression Medication^c^	No medication	12	Na	Na
	SSRI	4	Na	Na
	SNRI	1	Na	Na
	Other	4	Na	Na

*DYS*  dysphoric group, *CTRL*  control group, *SD*  standard deviation, *BDI-II*  Beck’s Depression Inventory, Second Edition, *DASS-A* Depression, Anxiety, Stress Scales, Anxiety scale (Lovibond and Lovibond 1995).

^a^Low = education level under high school, Medium = education level at high school or vocational school, High = education level at university.

^b^Depression severity based on participants self-report on their diagnosis or diagnosis given by project doctor. The diagnosis of depression was in accordance with the International Classification of Diseases and Related Health Problems, 10th Revision (ICD-10; World Health Organization, 2010) criteria. There is missing information related to disease severity from three participants, which is reported as severity unknown.

^c^*SSRI * selective serotonin reuptake inhibitor, *SNRI*  serotonin and norepinephrine reuptake inhibitor, *Other*  other antidepressant medication.

### Stimuli and experimental design

Amusing, sad and fearful movie clips were selected from the database of a previous study^49^. We selected movie clips from each emotional category (amusing, sad and fearful) based on the emotional feeling ratings that the participants of that study self-reported while watching them^49^. Movie clips with the most intensive emotional feelings were selected. Parts of the scenes were cut to avoid extremely strong depictions (e.g., violence) that were irrelevant to the target emotional categories. Because the dialogue in the movie clips was in English or French, and the participants were native Finnish speakers, all clips were presented without sound. Captions were not used, because they could affect the participants’ spatial attention to the visual scenes. The arousal score of each movie clip, as reported in Schaefer et al.^49^, is reported in Supplementary Table 1. According to the results of Schaefer et al.^49^, the mean arousal score of the selected movie clips is 5.478 for sad clips, 4.255 for amusing clips and 4.825 for fearful ones (per a 7-point scale, 1 = *no emotion at all*, 7 = *very intensive emotion*). To keep the total clip duration of each emotion category similar, different numbers of movie clips were used, totalling at 7 amusing, 5 sad and 6 fearful movie clips. For each emotional category, there was approximately 17 min’ worth of clips. Supplementary Table 1 presents descriptive information about the selected movie clips.

**Figure 5:**
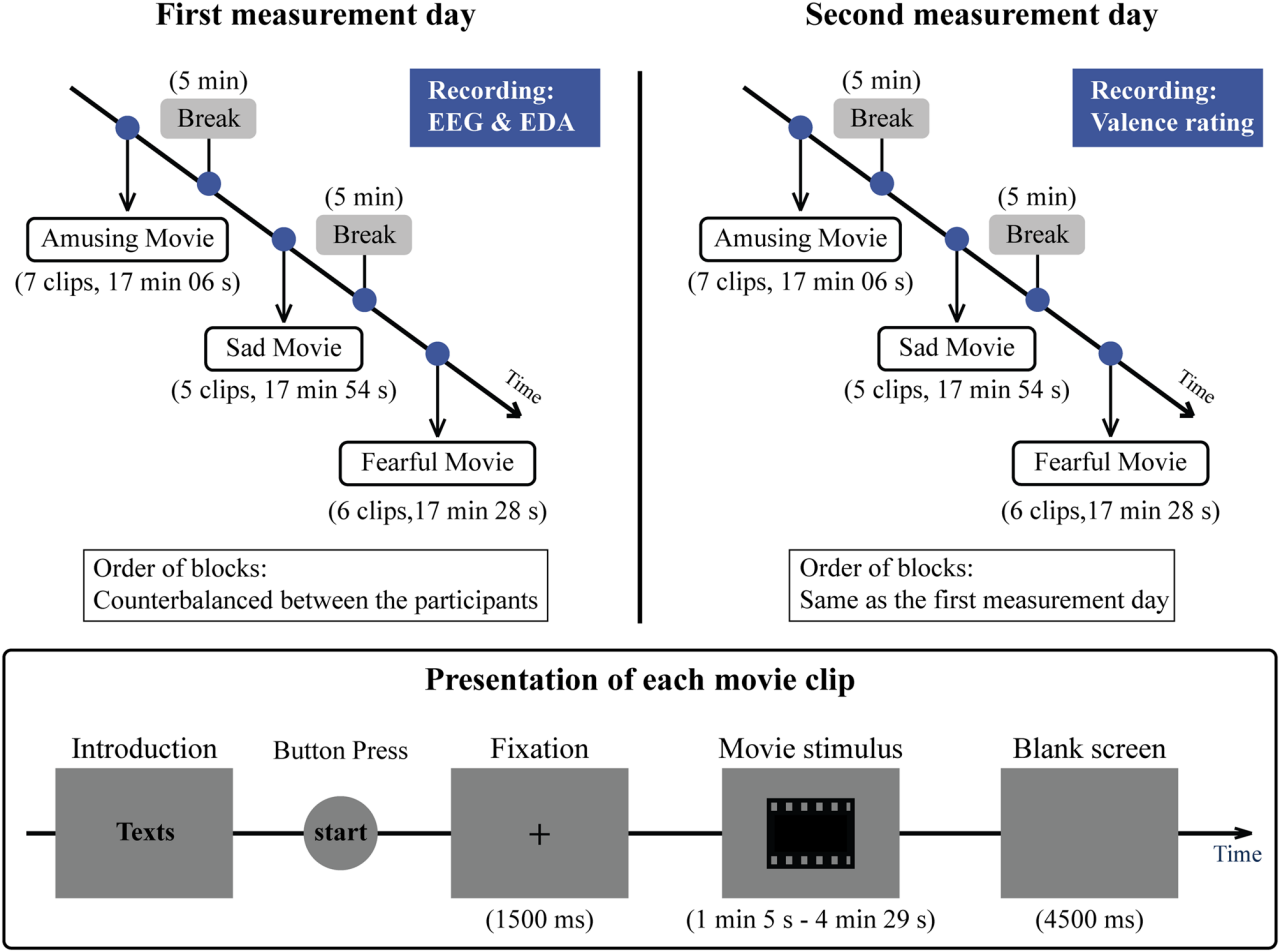
Experimental design. Psychophysiological and behavioural measurements were conducted for every participant on two separate days. Clips in the same emotional category were presented randomly. *EEG *electroencephalography; *EDA* electrodermal activity. EEG is not reported in the present paper.

The clips’ presentation was controlled with E-Prime 2.0 software (Psychology Software Tools, Inc., Sharpsburg, PA, USA). The movie clips were separated into three blocks corresponding to the three emotional dimensions (amusing, sad and fearful). The order of the blocks was counterbalanced between the participants. Within each block, clips in the same emotional dimension were presented randomly. Since we presented movie clips instead of whole movies, and the clips that were cut from the movies may be difficult to understand without context, we provided the participants a written introduction to the plot of the film before each clip. The text was written on the screen (Supplementary Table 2). After reading the introduction, the participants pressed a button to play the clip. Following a central fixation mark (1500 ms in duration), the movie clip was displayed at the centre of the screen at a resolution of 768 × 540 pixels. A blank screen was presented for 4500 ms after each movie clip. There was a 5-min break before the next block began. The experiment procedure is illustrated in Fig. 5.

Psychophysiological and behavioural measurements were conducted for every participant on two separate days. This procedure followed that of previous fMRI studies^20,53^ exploring inter-subject synchronization of neural responses to emotional stimulation, in which participants were rating their experience (valence/arousal) in a separate session from the brain activity recording. Here, it was also advisable to conduct the recordings of the brain activity and valence rating on separate days, because muscle activity (here caused by use of a joystick in valence rating task) can cause artefacts to electroencephalography (EEG) data^54^.For all participants, the psychophysiological measures were conducted on the first day and the rating task on the second day. We chose this order because it allowed the participants to naturally watch the movie clips during the psychophysiological measurements without thinking of their valence or memories from the rating task (which could have happened if they rated the clips first). Important to note, repeated viewing of emotional stimuli has only a small effect on the valence rating when stimuli contents are rated^55^.

On the first measurement day, EEG (these data are not reported here) and EDA data were collected as the participants watched the movie clips. The participants were seated in a sound- and electricity-shielded room with a dim light on the ceiling. A 23-inch screen (Asus VG236H, 1920 × 1080 pixels) was placed approximately 1 m in front of the participant to display the movie clips. Before the measurement began, the participants were aware that they were going to watch movie clips that may contain emotional scenes. However, the emotional categories were not revealed. The participants were instructed to sit still in a relaxed position during the movie screening, and they were told that they could end the experiment at any time.

On the second measurement day, the movie clips’ dynamic behavioural valence ratings (positive vs. negative) were recorded. By moving a joystick forward or backward, the participants evaluated the scenes’ valences on a continuous scale. Before the measurement began, the participants were instructed to practise for a few minutes so that they were familiar with the use of the joystick. There was a break after each movie clip, and the participants could start the rating for the next movie clip whenever they were ready. For each participant, the order of the blocks and movie clips was same as in the psychophysiological recordings.

### Recording and analysis of EDA data

#### EDA data recording

A continuous EDA was measured and amplified with a NeurOne system (Bittium Biosignals Ltd, Kuopio, Finland). Two disposable isotonic gel electrodes (Ag/AgCl, EL507, Biopac Systems, INC.) were placed on the participant’s nondominant hand (left hand). One of the electrodes was placed below the first digit (thenar eminence on the palm) and the other was attached on the same horizontal level below the fourth digit (hypothenar eminence on the palm). The electrodes were connected to a galvanic skin conductance module (Brain Products, Gilching, Germany) utilizing a constant current source with 0.5 V amplitude. The signal was recorded at a sampling rate of 1,000 Hz in DC mode, with a high cut-off of 250 Hz.

#### EDA data pre-processing

The EDA data were analysed using a MATLAB (R2016b) toolbox, Ledalab (Version 3.4.9). First, the sampling rate was reduced to 10 Hz. Next, adaptive smoothing was conducted to reject artefacts. Continuous decomposition analysis^56^ as performed to extract continuous phasic activity signals from the EDA responses as well. Then, the mean phasic EDA values were calculated for each movie clip and each participant. Lastly, for each participant, the phasic EDA in response to the movie clips was averaged for each movie type.

### Recording and analysis of valence rating

#### Valence ratings recording

The behavioural valence evaluation of each movie clip was recorded on the second day of measurement. The participants were instructed to evaluate the valence (positive, negative or neutral) of the scene content on a continuous scale by moving a joystick (Extreme 3D Pro, Logitech, Europe S.A.) forward or backward. The joystick was connected to E-Prime 2.0 software to control the presentation of movie clips and record the continuous valence ratings. The participants’ ratings were recorded at a 4 Hz sampling rate, ranging from − 1000 to 1000 on an artificial scale. To avoid possible response biases, for half of the participants, pushing the joystick forward rated a scene as positive, and pulling it backward rated the scene as negative. For the other half, this assignment was reversed.

#### Valence rating analysis

The behavioural valence ratings were analysed with a custom-made MATLAB (R2016b) script. Behavioural valence rating data were down-sampled to 1 Hz. To start, the mean valence rating value for each movie clip was calculated for all participants (Table 2). Based on the participants’ ratings, data from the movie clips were reselected for further analysis. For each amusing movie clip, the average valence rating should be positive; for each sad and fearful movie clip, the average valence rating should be negative. Since the average ratings of one of the amusing movie clips (Amusing 3 in Fig. 2) were negative, that clips’ data were excluded from the final analysis of both the EDA responses and behavioural ratings. The reason for the negative value of this presumably positive valence movie clip is unknown, but it is clear that this clip showed larger rating variance than any other (Table 2). After excluding this movie clip, the valence ratings were averaged first for each movie clip and then for each movie type per each participant.

**Table 2:** Mean values of the behavioural valence ratings for each movie clip (ratings ranging from − 1000 to 1000 on an artificial scale).

Movie type	Code of the movie	Mean	Standard deviation
Amusing	1	135.62	179.99
	2	42.07	141.26
	3	−46.47	264.11
	4	68.76	202.74
	5	78.95	196.37
	6	162.24	188.18
	7	16.79	250.85
Sad	1	−302.11	195.61
	2	−201.97	155.54
	3	−215.67	218.90
	4	−237.21	194.37
	5	−406.84	249.45
Fearful	1	−481.56	238.87
	2	−321.02	254.69
	3	−312.15	248.68
	4	−111.39	249.99
	5	−385.30	251.90
	6	−228.00	192.13

Amusing movie with code 3 was excluded from all the analysis due to negative mean value of the valence rating.

#### Valence rating ISC analysis

For each movie clip, we calculated the Pearson’s correlations of the time courses of the dynamic valence ratings between subject *k* and each other participant within the corresponding group (control or dysphoric). After this, one ISC value for each movie clip from each participant was obtained by averaging the individual correlations. Then, the ISC was obtained by averaging the correlation coefficient values of each movie clip type for each participant.

### Statistical analysis

All statistical analyses were conducted in IBM SPSS Statistics (Version 24, IBM Corporation, NY, USA).

The mean phasic EDA values, the average valence ratings, and the valence rating ISC for each movie type per each participant were analysed by using repeated measures ANOVA with Movie type (amusing vs. sad vs. fearful) as a within-subject factor and Group (control vs. dysphoric) as a between-subject factor, separately.

For the mean EDA values and the average valence ratings, whenever the main effect of Movie type emerged, paired samples t-tests with Bonferroni correction were conducted to compare the effect of each movie type. To investigate the possible effect of anxiety symptoms on the responses to Movie type, a separate analysis with DASS-A scores as a covariate was also performed by implementing an ANCOVA (Movie type × Group) for the whole group.

For the valence rating ISC, when an interaction effect (Movie type × Group) was statistically significant, comparisons based on independent t-tests were performed with a Bonferroni correction to investigate the difference in response to each movie type between the control and dysphoric groups. Repeated-measures ANOVAs and paired t-tests with Bonferroni corrections revealed the interaction effects within each group. To control for the possible effect of anxiety symptoms, a separate analysis with DASS-A scores as a covariate was also performed by implementing an ANCOVA for the dysphoric group. The effects of current medication status on valence ratings were explored by performing a repeated-measures ANCOVA with Medication (medicated vs. non-medicated) as a covariate for the dysphoric group.

Pearson’s correlation coefficients (two-tailed) were computed separately for each movie type to examine the relationship between phasic EDA (mean phasic EDA values) and depression symptoms (BDI-II scores), behavioural evaluations (mean valence rating values) and depression symptoms (BDI-II scores), and valence rating ISC and depressive symptoms (BDI-II scores) in the dysphoric and control groups. For the whole sample, Spearman’s rank correlation coefficients were calculated between the different measures, because the full sample’s BDI scores were not normally distributed.

For all results, we reported 95% CIs based on bootstrapping with 1000 permutations. The threshold for statistical significance was p < 0.050. Bonferroni-adjusted alpha levels less than 0.017 (0.050/3) were considered significant for *t*-tests investigating main or interaction effects. For the ANOVA results, *p-*values were reported based on the Greenhouse–Geisser correction, but the degrees of freedom were reported without correction. Partial eta-squared ${\eta }_{p}^{2}$ and Cohen’s d were given for the estimated ANOVA and *t*-test effect sizes, respectively. Cohen’s d was computed using pooled standard deviations^51^. Multiple correlations were controlled by applying a false discovery rate^57^ of 0.05.

### Ethical approval

This research follows the guidelines of ethical conduct and research reporting in accordance with the Declaration of Helsinki. The dataset (anonymous data including the main variables of the participants’ background information, EDA and behavioural ratings) and code that were developed for the data analysis are available to the community upon reasonable request.

## Supplementary Information

Supplementary Tables.

## References

[1] American Psychiatric Association. *American Psychiatric Association: Diagnostic and Statistical Manual of Mental Disorders Fifth Edition*. *Arlington* (2013).

[2] Beck, A. T. *Depression: Clinical, experimental, and theoretical aspects*. (Hoeber Medical Division, 1967).

[3] Beck AT (2008). The evolution of the cognitive model of depression and its neurobiological correlates. Am. J. Psychiatry.

[4] Peckham AD, McHugh RK, Otto MW (2010). A meta-analysis of the magnitude of biased attention in depression. Depress. Anxiety.

[5] Epp AM, Dobson KS, Dozois DJA, Frewen PA (2012). A systematic meta-analysis of the Stroop task in depression. Clin. Psychol. Rev..

[6] Caseras X, Garner M, Bradley BP, Mogg K (2007). Biases in visual orienting to negative and positive scenes in dysphoria: An eye movement study. J. Abnorm. Psychol..

[7] Kellough JL, Beevers CG, Ellis AJ, Wells TT (2008). Time course of selective attention in clinically depressed young adults: An eye tracking study. Behav. Res. Ther..

[8] Zhao Q (2015). Early perceptual anomaly of negative facial expression in depression: An event-related potential study. Neurophysiol. Clin..

[9] Zhang D, He Z, Chen Y, Wei Z (2016). Deficits of unconscious emotional processing in patients with major depression: An ERP study. J. Affect. Disord..

[10] Xu Q (2018). Automatic processing of changes in facial emotions in dysphoria: A magnetoencephalography study. Front. Hum. Neurosci..

[11] Ruohonen, E. M., Alhainen, V. & Astikainen, P. Event-related potentials to task-irrelevant sad faces as a state marker of depression. *Biol. Psychol.* **149**, 107806 (2020). 10.1016/j.biopsycho.2019.107806 31704201

[12] Leppänen JM, Milders M, Bell JS, Terriere E, Hietanen JK (2004). Depression biases the recognition of emotionally neutral faces. Psychiatry Res..

[13] Gollan JK, Pane HT, McCloskey MS, Coccaro EF (2008). Identifying differences in biased affective information processing in major depression. Psychiatry Res..

[14] Hasson, U. Intersubject synchronization of cortical activity during natural vision. *Science (80-. ).* **303**, 1634–1640 (2004). 10.1126/science.1089506 15016991

[15] Hasson U, Malach R, Heeger DJ (2010). Reliability of cortical activity during natural stimulation. Trends Cogn. Sci..

[16] Sachs, M. E., Habibi, A., Damasio, A. & Kaplan, J. T. Dynamic intersubject neural synchronization reflects affective responses to sad music. *Neuroimage* **218**, 116512 (2020). 10.1016/j.neuroimage.2019.116512 31901418

[17] Franchak JM, Heeger DJ, Hasson U, Adolph KE (2016). Free viewing Gaze behavior in infants and adults. Infancy.

[18] Lahnakoski JM (2014). Synchronous brain activity across individuals underlies shared psychological perspectives. Neuroimage.

[19] Nguyen M, Vanderwal T, Hasson U (2019). Shared understanding of narratives is correlated with shared neural responses. Neuroimage.

[20] Nummenmaa L (2012). Emotions promote social interaction by synchronizing brain activity across individuals. Proc. Natl. Acad. Sci. U. S. A..

[21] Lankinen K, Saari J, Hari R, Koskinen M (2014). Intersubject consistency of cortical MEG signals during movie viewing. Neuroimage.

[22] Lankinen K (2018). Consistency and similarity of MEG- and fMRI-signal time courses during movie viewing. Neuroimage.

[23] Dmochowski JP, Sajda P, Dias J, Parra LC (2012). Correlated components of ongoing EEG point to emotionally laden attention - A possible marker of engagement?. Front. Hum. Neurosci..

[24] Ki JJ, Kelly SP, Parra LC (2016). Attention strongly modulates reliability of neural responses to naturalistic narrative stimuli. J. Neurosci..

[25] Poulsen AT, Kamronn S, Dmochowski J, Parra LC, Hansen LK (2017). EEG in the classroom: Synchronised neural recordings during video presentation. Sci. Rep..

[26] Maffei A (2020). Spectrally resolved EEG intersubject correlation reveals distinct cortical oscillatory patterns during free-viewing of affective scenes. Psychophysiology.

[27] Chang WT (2015). Combined MEG and EEG show reliable patterns of electromagnetic brain activity during natural viewing. Neuroimage.

[28] Hasson U (2009). Shared and idiosyncratic cortical activation patterns in autism revealed under continuous real-life viewing conditions. Autism Res..

[29] Salmi J (2013). The brains of high functioning autistic individuals do not synchronize with those of others. NeuroImage Clin..

[30] Byrge L, Dubois J, Tyszka JM, Adolphs R, Kennedy DP (2015). Idiosyncratic brain activation patterns are associated with poor social comprehension in autism. J. Neurosci..

[31] Tu PC (2019). Reduced synchronized brain activity in schizophrenia during viewing of comedy movies. Sci. Rep..

[32] Yang, Z. *et al.* Individualized psychiatric imaging based on inter-subject neural synchronization in movie watching. *Neuroimage* **216**, 116227 (2020). 10.1016/j.neuroimage.2019.116227 31568871

[33] Guo CC, Nguyen VT, Hyett MP, Parker GB, Breakspear MJ (2015). Out-of-sync: Disrupted neural activity in emotional circuitry during film viewing in melancholic depression. Sci. Rep..

[34] Gruskin, D. C., Rosenberg, M. D. & Holmes, A. J. Relationships between depressive symptoms and brain responses during emotional movie viewing emerge in adolescence. *Neuroimage* **216**, 116217 (2020). 10.1016/j.neuroimage.2019.116217 PMC7958984 31628982

[35] Clark DA, Beck AT (2010). Cognitive theory and therapy of anxiety and depression: Convergence with neurobiological findings. Trends Cogn. Sci..

[36] Yiend J (2010). The effects of emotion on attention: A review of attentional processing of emotional information. Cogn. Emot..

[37] Dawson, M. E., Schell, A. M. & Filion, D. L. The electrodermal system. in *Handbook of Psychophysiology* 217–243. 10.1017/9781107415782.010 (Cambridge University Press, 2016).

[38] Berman MG (2011). Neural and behavioral effects of interference resolution in depression and rumination. Cogn. Affect. Behav. Neurosci..

[39] Koster EHW, De Lissnyder E, Derakshan N, De Raedt R (2011). Understanding depressive rumination from a cognitive science perspective: The impaired disengagement hypothesis. Clin. Psychol. Rev..

[40] Hautala, J., Loberg, O., Hietanen, J. K., Nummenmaa, L. & Astikainen, P. Effects of conversation content on viewing dyadic conversations. *J. Eye Mov. Res.* **9** (2016).

[41] Armstrong T, Olatunji BO (2012). Eye tracking of attention in the affective disorders: A meta-analytic review and synthesis. Clin. Psychol. Rev..

[42] Rantanen M (2021). Attentional bias towards interpersonal aggression in depression – An eye movement study. Scand. J. Psychol..

[43] Berenbaum H, Oltmanns TF (1992). Emotional experience and expression in schizophrenia and depression. J. Abnorm. Psychol..

[44] Sloan DM, Strauss ME, Quirk SW, Sajatovic M (1997). Subjective and expressive emotional responses in depression. J. Affect. Disord..

[45] Bylsma LM, Morris BH, Rottenberg J (2008). A meta-analysis of emotional reactivity in major depressive disorder. Clin. Psychol. Rev..

[46] Vuilleumier P (2005). How brains beware: Neural mechanisms of emotional attention. Trends Cogn. Sci..

[47] Fredrickson BL (2001). The role of positive emotions in positive psychology: The broaden-and-build theory of positive emotions. Am. Psychol..

[48] Bishop SJ (2007). Neurocognitive mechanisms of anxiety: An integrative account. Trends Cogn. Sci..

[49] Schaefer A, Nils F, Philippot P, Sanchez X (2010). Assessing the effectiveness of a large database of emotion-eliciting films: A new tool for emotion researchers. Cogn. Emot..

[50] Faul F, Erdfelder E, Lang A-G, Buchner A (2007). G*Power 3: A flexible statistical power analysis program for the social, behavioral, and biomedical sciences. Behav. Res. Methods.

[51] Cohen, J. *Statistical Power Analysis for the Behavioral Sciences*. *Statistical Power Analysis for the Behavioral Sciences*. 10.4324/9780203771587 (Routledge, 1988).

[52] Beck, A. T., Steer, R. A. & Brown, G. K. *Manual for the Beck Depression Inventory-II*. (Psychol. Corp., 1996).

[53] Nummenmaa L (2014). Emotional speech synchronizes brains across listeners and engages large-scale dynamic brain networks. Neuroimage.

[54] Luck, S. J. Basic principles of EPR recording. in *An Introduction to the Event-Related Potential Technique* 147–183 (Massachusetts Institute of Technology, 2014).

[55] Itkes O, Kimchi R, Haj-Ali H, Shapiro A, Kron A (2017). Dissociating affective and semantic valence. J. Exp. Psychol. Gen..

[56] Benedek M, Kaernbach C (2010). Decomposition of skin conductance data by means of nonnegative deconvolution. Psychophysiology.

[57] Benjamini Y, Yekutieli D (2001). The control of the false discovery rate in multiple testing under dependency. Ann. Stat..

