# Supplementary material for: Decreased intersubject synchrony in dynamic valence ratings of sad movie contents in dysphoric individuals
Source: Sci Rep. 2021 Jul 13;11:14419. doi: 10.1038/s41598-021-93825-1 (PMC8277793; doi:10.1038/s41598-021-93825-1)
Supplement: Supplementary file 1 — Supplementary Tables. [file 41598_2021_93825_MOESM1_ESM.pdf]

Decreased intersubject synchrony in dynamic valence ratings of sad movie contents in dysphoric individuals

Xueqiao Li, Yongjie Zhu, Elisa Vuoriainen, Chaoxiong Ye, & Piia Astikainen

Supplementary materials

In the supplementary materials, the descriptive information of selected movie clips is presented in the Supplementary Table 1. The introduction to the plot of each movie clip is shown in the Supplementary Table 2.

Supplementary Table 1 Descriptive information of selected movie clips.					
**Movie**	**Emotion**	**Code**	**Length**	**Arousal scoring**[a]	**Description of the scene**
City of angels	Sad	1	2'59"	5.15	Maggie dies in Seth's arms.
Dangerous mind	Sad	2	2'08"	5.25	Students in a school class are told that one of their classmates has died.
A perfect World	Sad	3	4'27"	5.78	Butch is gunned down, at the end of the movie.
The dead Poets Society (1)	Sad	4	3'51"	4.84	A schoolboy commits suicide.
Dead Man Walking	Sad	5	4'29"	5.87	The main character is put to death by lethal injection.
Les trois freres	Amusing	1	2'24"	4.36	One of the characters takes part in a TV game "the millionaire".
La cite de la peur	Amusing	2	2'13"	4.52	Three characters are at the dinner table and talk about the inquiry.
The visitors	Amusing	3	2'09"	3.55	Two men wearing medieval armors attack the postman's car.
When Harry met Sally	Amusing	4	2'45"	4.67	A female character fakes an orgasm in the restaurant, provoking Harry's embarrassment.
A fish called Wanda	Amusing	5	2'53"	4.04	The character gets undressed, waiting for his girlfriend. Unexpectedly, the owners of the house get into the house and discover him naked.
Le Pari	Amusing	6	1'47"	3.92	Lunch to celebrate father's birthday.
There is something about Mary (1)	Amusing	7	2'55"	4.02	Ted fights with a dog.
The Shining	Fear	1	4'15"	5.11	The character pursues his wife with an axe.
The Blair Witch Project	Fear	2	3'57"	4.95	Final scene in which the characters are apparently killed.
Scream 2	Fear	3	3'35"	4.81	A pursuit takes place in a school.
It	Fear	4	2'13"	4.69	A clown hidden in the sewer attracts a boy.
Copycat	Fear	5	2'23"	4.76	Monahan gets caught by a murderer in a toilet
Child's Play II	Fear	6	1'05"	4.63	Chucky beats Andy's teacher.

Note. The arousal scorings of the movie clips applied here were reported in Schaefer et al.[1], which showed that the mean values of sad movie clips were the highest (by using a 7-piont scale, 1 = "no emotion at all", 7 = "very intensive emotion", sad: 5.478, amusing: 4.255, fearful: 4.825).

Supplementary Table 2 Introduction to the plot of each movie clip. The introduction was presented in Finnish in the measurement.

Movie	English	Finnish
City of angels	Seth and Maggie have finally been together. The next morning, as Seth is in the shower, Maggie rides her bicycle to a local store. On her way back, happy and fulfilled.	Seth ja Maggie ovat vihdoin olleet yhdessä. Seuraavana aamuna Sethin ollessa suihkussa, Maggie pyöräilee paikalliseen kauppaan. Paluumatkallaan, onnellisena ja tyytyväisenä …
Dangerous mind	Emilio is one of LouAnne's student. When LouAnne discovers that Emilio's life is in danger, she tries to protect him. She advises him to seek help from Principal Grandey. The next day, Emilio visits Grandey, but Grandey instantly dismisses him because he neglected to knock on Grandey's door before entering his office. Feeling rejected, Emilio leaves the school and is subsequently killed by his enemy.	Emilio on yksi LouAnnen oppilaista. Saadessaan selville, että Emilion elämä on vaarassa, yrittää LouAnne suojella häntä. LouAnne ohjaa Emilion rehtori Grandeyn puheille. Seuraavana aamuna Emilio käykin rehtorin työhuoneessa, mutta unohtaa koputtaa oveen ennen sisään astumistaan. Tämän vuoksi Grandey käännyttää hänet heti pois. Emilio poistuu koulusta koettuaan tulleensa hylätyksi ja joutuu myöhemmin vihollisensa murhaamaksi.
A perfect World	After escaping from a Huntsville prison, convict Butch and his partner Terry kidnap a young boy, Philip, and flee across Texas. As they travel together, Butch and Philip discover common bonds and suffer the abuses of the outside "Perfect World." In pursuit is Texas Ranger "Red" Garnett and criminologist Sally Gerber. Red's team surrounds the place where Phillip and Butch are situated, the latter sending the boy away to his mother, who is with Red's team.	Karattuaan Huntsvillen vankilasta, vanki Butch ja hänen seuralaisensa Terry kidnappaavat nuoren pojan, Philipin, ja pakenevat läpi Texasin. Yhdessä matkatessaan, Butch ja Philip huomaavat olevansa keskenään samanlaisia ja kärsivänsä ulkopuolisen, täydellisen maailman pahuudesta. Takaa-ajossa ovat osallisena Texasin ratsupoliisi ”Red” Garnett sekä kriminologi Sally Gerber. Redin tiimii saartaa alueen, jossa Philip ja Butch ovat. Lopulta Butch lähettää Philipin äitinsä luokse, joka on Redin tiimin mukana.

The dead Poets Society (1)	Neil Perry, although exceedingly bright and popular, is very much under the thumb of his overbearing father. Neil, along with their other friends, meets Professor Keating, their new English teacher, who tells them of the Dead Poets Society, and encourages them to go against the status quo. However, Neil is unable to find the courage to stand up to his father.	Neil Perry on erittäin fiksu ja suosittu, mutta myös hyvin paljon määräilevän isänsä tossun alla. Neil tapaa ystäviensä kanssa uuden englannin opettajansa, professori Keatingin, joka kertoo heille Kuolleiden runoilijoiden seurasta ja rohkaisee heitä vastustamaan nykyisiä olosuhteita. Neil ei kuitenkaan löydä tarpeeksi rohkeutta vastustaa isäänsä.
Dead Man Walking	Matthew Poncelet has been in prison for six years, awaiting his execution after being sentenced to death for killing a teenage couple.As the day of his execution comes closer, Poncelet asks Sister Helen, with whom he has corresponded, to help him with a final appeal. After many visits, she establishes a special relationship with him. Sister Helen’s application for a pardon is declined. Poncelet asks Sister Helen to be his spiritual adviser through the day of execution, and she agrees.	Matthew Poncelet on ollut vankilassa kuusi vuotta. Hänet tuomittiin kuolemaan teiniipariskunnan taposta ja nyt hän odottaa tuomionsa toteutumista. Kuolemantuomipäivän lähestyessä Poncelet pyytää kirjeystäväänsä, sisar Heleniä, auttamaan häntä viimeisen vetoomuksensa kanssa. Usean vierailukerran jälkeen, Helenin ja Ponceletin välille muodostuu erityinen suhde. Sisar Helenin vetoomus armahduksesta ei kuitenkaan mene läpi. Poncelet pyytää Heleniä hengelliseksi avuksi kuolemantuomiopäivänään ja Helen suostuu tähän.
Les trois freres	Three half-brothers are reunited at their mother's funeral. After being told of their inheritance they quickly spend the money, only to find out that they will not receive it after all. The brothers try to work out what to do and decide to participate in a TV game.	Kolme velipuolta tapaa toisensa pitkästä aikaa äitinsä hautajaisissa. Kuultuaan heille kuuluvasta perinnöstä kuluttavat he nopeasti perintörahansa, mutta kuulevatkin sitten, etteivät tulekaan saamaan perintöä. Veljekset miettivät, kuinka ratkaisisivat tilanteen ja päättävät osallistua TV-peliin.
La cite de la peur	Conversation between three characters at a dinner table.	Kolmen henkilön välinen keskustelu päivällispöydässä.
The visitors	A medieval nobleman and his squire are accidentally transported to contemporary times by a senile sorcerer.	Keskiaikainen aatelismies ja hänen aseenkantajansa siirtyvät vahingossa nykyaikaan vanhuudenhöperön velhon loihtimana.

When Harry met Sally	Harry and Sally have known each other for years, and are very good friends, but they fear sex would ruin the friendship. Harry and Sally are having dinner together.	Harry ja Sally ovat tunteneet toisensa vuosia ja ovat todella hyviä ystäviä, mutta pelkäävät, että seksi pilaisi heidän ystävyytensä. Harry ja Sally syövät päivällistä yhdessä.
A fish called Wanda	Sexy American diamonds lover Wanda and her boyfriend Otto are in England to plot alongside George and Ken the robbery of a diamond collection. Wanda and Otto want the stolen diamonds for themselves, and try to doublecross others for the loot. However, George has already moved the diamonds to another secret place. Wanda thinks the best way to find out is by getting close to George's lawyer - Archie. Now, Wanda and Archie are at house.	Viehättävä amerikkalainen timantteja rakastava Wanda suunnitteli timantti kokoelman ryöstön rikoskumppaneidensa kanssa. Hän kuitenkin tahtoo timantit kokonaan itselleen ja pyrkii varastamaan ryöstösaaliin myös kumppaneiltaan. George, yksi rikoskumppaneista, on siirtänyt timantit salaiseen paikkaan. Wanda ajattelee, että paras tapa selvittää timanttien olinpaikka on lähestyä Georgen asianajajaa Archieta. Nyt Wanda ja Archie ovat toisen kotona.
Le Pari	Bernard is a teacher in the suburbs and lives with Victoria. Didier is a wealthy Parisian pharmacist and is married to Murielle, Victoria's sister. While the former drives a rusty car, the latter drives a black Mercedes. Both brothers-in-law are complete opposites and hate each other. They are having a lunch to celebrate father’s birthday.	Bernard toimii opettajana lähiössä ja asuu yhdessä Victorian kanssa. Didier on varakas pariisilainen apteekkari ja on naimisissa Muriellan, Victorian sisaren, kanssa. Bernard ajaa ruosteisella autolla, kun taas Didierillä on musta Mercedes. He ovat toistensa vastakohtia ja vihaavat toisiaan, ja tässä he ovat juhlimassa appensa syntymäpäivää lounaalla.
There is something about Mary (1)	Ted gets a chance to meet up with his dream girl, Mary, from high school, even though his date with her back then was a complete disaster. Ted is having a conversation with Mary.	Ted saa mahdollisuuden tavata lukioaikaisen unelmien tyttönsä Maryn. Lukioaikana hänen treffinsä Maryn kanssa olivat täydellinen katastrofi. Ted keskustelee Maryn kanssa.

The Shining	A family heads to an isolated hotel for the winter where an evil and spiritual presence influences the father into violence, while his psychic son sees horrific forebodings from the past and of the future.	Perhe matkustaa eristyksissä olevaan hotelliin talveksi, jossa yliluonnollisen pahan läsnäolo saa perheen isän käyttäytymään väkivaltaisesti, samalla kun hänen meedio poikansa näkee kauhistuttavia pahoja enteitä menneestä ja tulevasta.
The Blair Witch Project	Three film students travel to a Maryland forest to film a documentary on the local Blair Witch legend	Kolme elokuva-alan opiskelijaa matkustavat Marylandin metsään tekemään dokumenttielokuvaa paikallisen noidan legendasta.
Scream 2	Two years after the events of Scream, Sidney Prescott and Randy are attending Windsor college. They are trying to get on with their lives.	Kaksi vuotta Screamin tapahtumien jälkeen Sidney Prescott ja Randy käyvät Windsorin yliopistoa ja yrittävät vain jatkaa elämäänsä.
It	In 1960, a group of social outcasts who are bullied by a gang of greasers led by Henry Bowers are also tormented by an evil demon who can shape-shift into a clown and feed on children's fears and kill them.	On vuosi 1960 ja hyljeksittyjen poikien ryhmää jota hätyyttää kiusaajien jengi Henry Bowersin johdolla, piinaa myös paha demoni, joka kykenee muuttamaan muotoaan pelleksi, saa voimansa lasten peloista ja pyrkii tappamaan heidät.
Copycat	Helen, an agoraphobic psychologist and M.J., a female detective work together to take down a serial killer who copies serial killers from the past. During the search for the serial killer, M.J. discovers that the killer has kidnapped Helen and taken her back to the restroom of the lecture hall. M.J. is getting there.	Helen, agorafobinen psykologi ja M.J., naisetsivä tekevät yhteistyötä saadakseen kiinni sarjamurhaajan, joka kopioi menneisyyden sarjamurhaajia. Murhaajan etsintöjen aikana M.J. huomaa murhaajan kaapanneen Helenin ja vieneen tämän takaisin luentosalin wc:hen. M.J. on matkalla Helenin luo.

Child's Play II	When Andy’s mother is handed over to a psychiatric hospital, the young boy is placed in foster care, as is the Chucky doll who is determined to take Andy’s soul. Chucky ends up on the bus to Andy’s school and Andy’s teacher finds the obscenities written by Chucky in this workbook. The teacher believes Andy is to blame for this and forces him to be left alone in the classroom as punishment and locks Chucky in the closet.	Kun Andyn äiti luovutetaan psykiatriseen sairaalaan joutuu nuori poika sijaishoitoon, samoin kuin Chucky nukke, joka on päättänyt vallata Andyn sielun. Chucky päätyy bussilla Andyn kouluun ja Andyn opettaja löytää tämän työvihkosta Chuckyn kirjoittamat rivoudet. Opettaja uskoo Andyn olevan tähän syyllinen ja pakottaa hänet jäämään yksin luokkahuoneeseen rangaistuksena ja lukitsee Chuckyn komeroon.

References

1. Schaefer, A., Nils, F., Philippot, P. & Sanchez, X. Assessing the effectiveness of a large database of emotion-eliciting films: A new tool for emotion researchers. *Cogn. Emot.* **24**, 1153–1172 (2010).
